# How to Best Develop and Deliver Generic Long-Term Condition Rehabilitation Programmes in Rural Settings: An Integrative Review

**DOI:** 10.3389/fresc.2022.904007

**Published:** 2022-06-21

**Authors:** Amanda Wilkinson, Chris Higgs, Tim Stokes, Jack Dummer, Leigh Hale

**Affiliations:** ^1^Centre for Health, Activity and Rehabilitation Research, School of Physiotherapy, University of Otago, Dunedin, New Zealand; ^2^Department of General Practice and Rural Health, University of Otago, Dunedin, New Zealand; ^3^Department of Medicine, University of Otago, Dunedin, New Zealand

**Keywords:** community, long-term conditions, rehabilitation programmes, rural, telehealth

## Abstract

People living rurally frequently experience health disparities especially if living with a long-term condition (LTC) or multi-morbidity. Self-management support is a key component of LTC management and commonly included in rehabilitation programmes to enhance ability to self-manage health and encourage physical activity. Such programmes are however often condition focussed and despite evidence for their effectiveness, are not always feasible to deliver in rural settings. Generic programmes are arguably more optimal in the rural context and delivery can be face to face or remotely (via telehealth). The aim of this explorative integrative review was to collate and present international evidence for development, delivery, integration, and support of community-based, generic LTC group rehabilitation programmes delivered rurally in person, or remotely using telehealth. Electronic databases were systematically searched using MeSH terms and keywords. For inclusion, articles were screened for relevance to the aim, and practical information pertaining to the aim were extracted, charted, and organized deductively into themes of Development, Delivery, Integration, and Support. Within each theme, data were synthesized inductively into categories (Theory, Context, Interpersonal aspects, and Technology and Programme aspects). Fifty-five studies were included. Five studies contributed information about community based programmes delivered *via* the internet. *Development* was the only theme populated by information from all categories. The theme of *Support* was only populated with information from one category. Our review has drawn together a large body of diverse work. It has focused on finding practical information pertaining to the best ways to develop, deliver, integrate, and support a community-based generic rehabilitation programme for people living with long-term health conditions, delivered rurally and/or potentially *via* the internet. Practical suggestions were thematically organized into categories of theory, context, interpersonal aspects, and technology and programme aspects. While the findings of this review might appear simple and self-evident, they are perhaps difficult to enact in practice.

## Background

Long-term health conditions (LTCs) are any ongoing, long term or recurring health conditions (>6 months) (1). LTCs impact significantly on a person, their family and their wider community (2). Self-management support is a key component in the health care of people living with LTCs. Rehabilitation programmes are important in the management of LTCs and usually comprise of components of exercise and education, with a focus on self-management support so that the person can learn to live and manage their condition (3). A systematic review identified the key features of LTC rehabilitation programmes as being of 4–8 weeks in length, and include education on symptom management, exercise, time to develop and embed self-management skills and self-efficacy, and led by health professional/s together with lay or peer leaders (4). In previous work, we identified that the viability and sustainability of rehabilitation programmes may be contingent on a “closer to home” generic approach catering for people with more than one long-term condition (5). Further, building relationships, not just between the healthcare providers and people attending with LTCs, but between both these groups and the wider community are vital to enable and maintain participation (5, 6). These factors may become even more crucial when working in rural or remote communities to enable health equity and promote supported self-management in a wider context.

Health care for people living with LTCs, particularly in secondary care, has largely been driven by models relating to one condition (7), whereas the increase in prevalence of multi-morbidity demands more complex models of care (8–10). In terms of rehabilitation programmes, LTCs are mostly dealt with as single conditions, for example a current large undertaking by the World Health Organization and Cochrane Rehabilitation is developing a “WHO Package of Rehabilitation Interventions” (11). This project is developing rehabilitation guidelines for 20 separate health conditions as opposed to grouping conditions together by functional outcomes (12). For many LTCs, one of the mainstays of management is exercise or physical activity. Whilst specific conditions have specific exercise guidelines [for example, cardiac rehabilitation, (13) pulmonary rehabilitation (14)], in reality, the optimal exercise regimen (i.e., exercise type, intensity, duration, and frequency) is remarkably similar across LTCs (3). The benefits of condition-specific rehabilitation include high evidence in improving exercise capacity, symptoms, health related quality of life, and reducing hospitalization (15, 16). The challenges to condition specific rehabilitation include having available healthcare professional specialists to run it, sufficient class attendees, and the nonsensical approach of people living with multiple morbidity attending specific rehabilitation programmes for each condition they are diagnosed with. Many LTC rehabilitation classes are delivered from a secondary care (hospital) setting, and thus become, and are perceived to be, “medicalised” in nature (16, 17). Conducting generic, as opposed to single condition, LTC rehabilitation programmes is an emerging concept.

Despite robust evidence for benefit of LTC rehabilitation (15), in many countries in the world attendance at rehabilitation programmes is hindered by many factors, and particularly in urban or remote areas (18–21). Inequities in healthcare provision are compounded by distance from health services, reduced access to primary and specialist care clinicians, and reduced socio-economic status and low health literacy (21–23). In New Zealand, rural towns have the lowest socioeconomic status, highest proportion of Māori, and the highest avoidable and amenable mortality rates. Telehealth (delivery of healthcare when patients and healthcare professionals are in separate locations) (24) may be a possibility for delivering healthcare. Delivering generic community-based rehabilitation programmes in rural areas in person or by using telehealth may provide more equitable access to services beneficial to improving the health and wellbeing of those living with long-term conditions, and to a population in need of such a service (25). However challenges remain to using both approaches and in particular telehealth (such as equitable access to the internet, cost of technology, security breaches, technological and software limitations, changes in patient expectations and engagement, difficulty in maintaining therapeutic relationship and reading non-verbal cues) (26, 27).

Nevertheless, informal consultation suggests that the potential benefits of offering a generic programme in person or by using telehealth include (i) healthcare delivered closer to home to remove some of the barriers for consumers through using community facilities (church and community halls, local gyms) set up for in person or telehealth delivery of a proactive programme enabling people to take control of their own health and make healthy choices; and (ii) potential reduced requirements for acute care by keeping people fitter and independently living at home. Whilst Mulligan, Wilkinson (4) identified the components of a generic community rehabilitation programme for people with LTCs, these were not specific to a rural setting or indeed one delivered using telehealth. This explorative integrative review thus sought information pertaining to international practice in developing, delivering, sustaining, and supporting a community-based, generic LTC group rehabilitation programme delivered rurally in person or remotely using telehealth.

## Methods

As our review was exploratory, we employed an integrative review method as it has the broadest type of search remit, allowing for multiple study types and methodologies to be included in the review (28, 29). The inclusion of such diverse literature provides the opportunity to gather a greater scope of articles and gain a deeper understanding of the topic to answer the research question more effectively.

An initial search of Google Scholar was undertaken to explore potential search terms relating to the research question. After discussion with a subject librarian and individual research team members, and exploration of OVID Medline, a table of potential search terms and their associated MeSH terms was developed (see Table 1). A methodical search (30) of Google Scholar, SCOPUS, TRIP, Cochrane, EBSCO (CINAHL), JBI, OVID (Medline, Embase, Emcare, Psychinfo) and SCiello was then undertaken (November-December 2020) using combinations of MeSH terms and keywords (as appropriate for each database). All searches used Boolean operators “AND” and “OR”. Discussion with experts in the field and searching of relevant journals (such as Journal of Rural Health) were also undertaken to generate further potential articles. Reference lists of potential articles were not searched for further potential articles.

**Table 1:** Generic database list of MeSH and keyword search terms.

**MeSH terms**	**Chronic disease**	**Adult**	**Self-management**	**Community, rural**	**Exercise**	**Education**	**Program, viability, acceptability**	**Telemedicine**
Keywords	Chronic disease Long-term condition/s Chronic illness Multimorbidity	Adult Middle aged Aged Aged, 80 and over Socioeconomic factors	Self-care Self-management Self-management Support.mp	Communit*.mp Neighborhood*.mp Rural health Rural health services Center, rural health Centers, rural health Health center, rural Health centers, rural Health service, rural Health services, rural Rural health center Rural health centers Rural health service Rural health services Service, rural health Services, rural health	Exercise Activities, physical Activity, physical Acute exercise Acute exercises Aerobic exercise Aerobic exercises Exercise Exercise, acute Exercise, aerobic Exercise, isometric Exercise, physical Exercise training Exercise trainings Exercises Exercises, acute Exercises, aerobic Exercises, isometric Exercises, physical Isometric exercise Isometric exercises Physical activities Physical activity Physical exercise Physical exercises Training, exercise Trainings, exercise Exercise therapy Exercise, rehabilitation Exercise, remedial Exercise therapies Exercise therapy Exercises, rehabilitation Exercises, remedial Rehabilitation exercise Rehabilitation exercises remedial Exercise Remedial exercises Therapies, exercise Therapy, exercise	Health promotion Health behavior Healthy lifestyle Education Activities, educational Activity, educational Education Educational activities Educational activity Literacy program Literacy programs Program, literacy Program, training Programs, literacy Programs, training Training program Training programs Workshop Workshops Patient education as topic Education, patient education of patients Patient education Patient education as topic Health Education Community health education Education, community health Education, health Health education Health education, community	Program evaluation Social validity, research	Telemedicine Health, mobile Mobile health Telehealth Telemedicine ehealth Mhealth

Potential articles were title and abstract screened for relevance to the research question and had to include key terms of “Chronic illness/disease/long-term conditions”, “Adult”, “Community, rural” and “Self-management” combined with terms of “Exercise”, “Education”, “Program, viability, acceptability”, and “Telemedicine” as appropriate to the individual databases (see Table 1). Articles were not included if they discussed home-based interventions delivered to one person, were delivered in a hospital or outpatient setting, included children/young people, or were not written in English. Extracted data required relevance to the research question with a focus on practical information pertaining to themes of *development, delivery, integration*, and ongoing *support* of a community-based, generic rehabilitation programme for people living with long-term health conditions (irrespective of the type of condition) delivered rurally and/or potentially *via* the internet (see Table 2 for definitions of themes). The full article was read if it were unclear in the abstract if it were relevant to the research aim. One author (AW) was responsible for decisions around suitability of articles for inclusion. Given the nature of this explorative integrative review and expected capture of publications with diverse study designs, included studies were not quality appraised.

**Table 2:** Definition of themes.

**Themes**	**Definition**
Development	Information describing how a programme was created and grown, and explanation about content of programme
Delivery	Practical information explaining how programme was delivered, and or made accessible to people in their community
Integration	Explanation about how programme was incorporated into the community (the setting and structures of the community) and into people's lives
Support	Information provided about how the programme was maintained in a community

Data analysis, undertaken by one author (AW), involved extracting information pertaining to author, year, country or paper methodology, aim, and “demographics” of the study, review or report. This information was tabulated into an overall summary of included studies (see Table 3). Data pertaining to practical information about *how to* develop, deliver, integrate, and support a remotely delivered programme was then extracted from included studies. Through discussion and consensus by two authors (AW, LH) this information was deductively organized into “themes” (development, delivery, integration and support). These themes were derived from the research question, which was informed by multiple collaborative conversations with community stakeholders and modified from the RE-AIM framework (54) Data within each “theme” was then inductively (84) synthesized, again by the two authors, into five categories.

**Table 3:** Summary of included studies.

**Author, year, country or methodology**	**Aim**	**Demographics of study/review/report**	**Contributed information**			
			**to themes of:**			
			**Development**	**Delivery**	**Integration**	**Support**
Ball et al. (31); Australia; Primary study	Examine the reach, retention, sociodemographic and health characteristics, physical activity levels and motivations for joining and remaining in the Heart Foundation Walking programme	*n* = 22,416 people aged 15+		✓	✓	
Banbury et al. (32); Literature review	Determine feasibility, acceptability, effectiveness, and implementation of group video conferencing of education or social support or both into the home setting	*n =* 17 healthcare professionals (family practices, primary care organizations, generalist community health service and tertiary providers; patient education or social or mental health support)*.	✓	✓		
Banbury et al. (33); Australia; Primary study	Co design, test and evaluate a health chronic disease self-management and social support intervention delivered *via* group video conferencing into the home	*n =* 112 older people (*n =* 52 intervention, *n =* 60 control)	✓	✓		
Barker et al. (16) Australia Primary study	Feasibility/pilot of rehabilitation programme for people with multi-morbidity versus usual care	*n =* 16 people with multimorbidity (*n =* 8 intervention group)	✓			
Barnidge et al. (34); Rural south east Missouri, USA; Primary study	Describes how authors used regional partnership to leverage resources and enhance environmental and policy initiatives to improve nutrition and physical activity for rural people with long-term conditions	*n =* 30 community partners from 12 Healthier Missouri Communities counties	✓		✓	
Bradford et al. (26); Literature review	To describe telehealth services in rural Australia and identify factors associated with sustained success	*n =* 116 articles describing 72 services			✓	
Brown et al. (35); Rural primary care clinics, USA; Primary study	Proof of concept study describing a telemedicine weight management programme	*n =* 86 patients with obesity	✓		✓	
Brundisini et al. (36); Literature review	Identify advantages and disadvantages rural patients with chronic disease experience when accessing rural and distant care	*n =* 12 primary qualitative studies			✓	
Burford et al. (37); Australia; Primary study	To design 6 invitations for patients with T2DM to explore *via* their tablets	*n =* 11 (7 doctors, 1 specialist, 2 nurses, 1 practice manager)	✓		✓	✓
Cheng et al. (38); Literature review	Explore the role of eHealth literacy and user involvement in developing eHealth interventions for socially disadvantaged groups	*n =* 51 studies (48 interventions)	✓			
Coghill et al. (39); Rural health units, Canada; Primary study	Explore chronic disease prevention interventions that have or are being implemented which address built environment related to PA and impact of interventions	*n =* 12 rural public health practitioners and managers	✓			
Dalhberg et al. (40); Literature review	Explored perspectives of Indigenous Australians around physical activity barriers and facilitators	*n =* 8 studies	✓			
Del Bello-Haas et al. (41); Rural Saskatchewan, Canada; Primary study	Examine demand, acceptability, practicality, and implementation of 4-wk telehealth exercise intervention; rural community dwelling people (with dementia) and their caregivers;	Survey (*n =* 77; *n =* 42 people; *n =* 35 caregivers); *n =* 2 patient-caregiver dyads participated in programme and interviews		✓		
Dent et al. (42); Rural Australia	Implement and evaluate a population health intervention using Co-KT framework	People with musculoskeletal conditions	✓		✓	
Diaz-Skeete et al. (43); Ireland; Primary study	Explore barriers and facilitators to adoption of eHealth technology and remote monitoring systems in community and home for cardiac care	*n =* not stated; clinicians, academic researchers, technologists, patient advocates, policy makers and representatives from health service	✓	✓		✓
Dobkin (44); Literature review	Synthesis of current opinion	Self-management training should be an explicit component of rehabilitation care and clinical trials	✓			
Draper et al. (45); Low income rural communities, South Africa; Primary study	Assess the process of implementation of Chronic disease prevention Discovery Healthy Lifestyle Programme to identify facilitators and barriers.	*n =* 45; Teachers, nurses, and community volunteers			✓	
Dye et al. (46); USA; Primary study	Description of 8-week community hypertension self-management programme implemented by trained volunteers;	*n =* 185; patients	✓	✓	✓	
Evans and Buck (47); England; Primary study	The Kings Fund – tackling multiple unhealthy risk factors	Rural and urban case studies from NHS; Used Michie et al. (48) theory for behavior change (COM-B)*	✓			
Field et al. (49); Remote Papua New Guinea; Primary study	Describe the monitoring and evaluation (M&E) conducted for the Community Mine Continuation Agreement (CMCA) Middle and South Fly Health Program	Offers practical solutions from lessons learned	✓		✓	
Garrubba and Melder (50); Literature review	Identified evidence for guiding innovative thinking and planning in the development of a community-based health service for future healthcare needs of consumers	*n =* 12 government reports, commissioned papers, health service reports and white papers from international and Australian sources			✓	
Gavarkovs et al. (51); Canada, rural; Primary study	Obtain the perspective about barriers to effective recruitment and participation of men in chronic disease self-management programme.	*n =* 10 programme delivery staff		✓		
Gavarkovs et al. (52); Rural to large population areas Canada; Primary study	Examine the perceived physical activity–related barriers and facilitators experienced by men with chronic diseases living in rural areas	*n =* 149 men, aged 18–85+			✓	
Glasgow and Estabrooks (53); Theoretical paper	To make RE-AIM (Reach, Effectiveness, Adoption, Implementation, Maintenance) transparent.	Describes processes used and provides questions for internal and external validity in research. RE-Aim is a planning and evaluation framework for use in community and clinical settings, translational research public health and policy.	✓			
Glasgow et al. (54); Theoretical paper	Discusses evolution, application, and challenges of using RE-AIM.	RE-AIM encourages expanded focus on multiple factors that impact public health (QoL, or unintended consequences). Encourages pragmatic use of key dimensions rather than all elements.	✓			
Glasgow et al. (55); Theoretical paper	To summarize key issues in the eHealth field from an implementation science perspective and to highlight illustrative processes, examples, and key directions to help more rapidly integrate research, policy, and practice.	Describes evolving practical learnings	✓		✓	
Heath et al. (56); Review of reviews	Identify effective, promising, or emerging physical activity interventions from around the world; children, adolescents, or adults without disease	*n =* 100 studies; classified according to campaigns and informational approaches, behavioral and social approaches, and environmental and policy approaches.			✓	
Hege et al. (57); Rural USA; Primary study	Exploring environmental barriers to active living	*n =* 16 rural towns and townships across seven counties			✓	
Ignatowicz et al. (58); Review of reviews	Use of internet videoconferencing for consultations between HCPs and patients with LTCs in their own home	*n =* 35 reviews	✓			
Jaglal et al. (59); Rural and remote Canada; Primary study	Examine if access to telehealth self-management programme improves self-efficacy, health behaviors, and health status and if there are differences between delivery models - single site and multiple site	*n =* 213 chronically ill adults		✓		
Joseph and Melder (60); Rapid review	Synthesize evidence about efficacy, cost, sustainability and appointment attendance and use of video for clinical consultations to inform development of new video conferencing service	*n =* 7 studies; clinical areas of diabetes, nephrology, oncology, hematology, genetics, pain management, medication review, infectious disease			✓	
Khan et al. (61); Literature review	Assess effectiveness and safety of tele-rehabilitation for improved outcomes	*n =* 9; multiple sclerosis			✓	
Knox et al. (62); Wales; Primary study	Assessment of feasibility, safety, and effectiveness of virtual pulmonary rehabilitation programme (VIPAR)	*n =* 21 patients with stable lung disease	✓	✓		
Kuluski et al. (63); Ontario, Canada, and New Zealand; Primary study	Describe attributes of care that are important to older people with multi-morbidities (2 or more chronic conditions), and their caregivers	*n =* 172 patients (65 years+) and caregivers from nine case study sites	✓			
Maddox et al. (64); Report	Report on the drought related mental health needs of farmers in rural and remote Australia	Reorientation process including development and use of program logic model (PLM) to facilitate Rural Adversity Mental Health Program implementation			✓	
May (65); Theoretical paper	Presents a theory of implementation and embedding of innovations	Use shared decision making (SDM) to show how Normalization Process Theory (NPT) could be used in a clinical encounter or as people try to embed healthcare innovations into practice			✓	
May and Finch (66); Theoretical paper	Outlines Normalization Process Theory (NPT)	Contributes to discussion on how “something” becomes normalized in an individual's life, community, or system.			✓	
Mulligan et al. (4); Literature review	Identify core programme components and clinically meaningful measures for generic chronic condition rehabilitation programmes	*n =* 15 studies (RCTs); adults	✓	✓		
NZ Govt, Internal Affairs (67); Literature review	Provide an overview of approaches to grant making for community development	Review of projects from USA, Canada, and England	✓		✓	
Oliver et al. (68); Theoretical paper	Describe methods used, facilitators, barriers and goals for involving consumers in a needs-led health research programme (Health technology assessment).	Analysis of policy and procedure documents, minutes, agendas, letters, observations of panel and staff meetings	✓			
Oliver et al. (69); Report	Identify barriers to and facilitators of involving consumers in meaningful participation in research identification and prioritization in UK	*n =* 286 documents	✓		✓	
Peel et al. (70); Report	Reports development of new British Lung Foundation 12-month remote telephone service (health coaching) for inactive people with lung conditions.	Physical activity programme delivered according to the key stages of the Behavior Change Wheel	✓			
Pelletier et al. (71); Literature review	Map literature describing implementation of physical activity interventions in rural, and/or remote communities	*n =* 12 studies			✓	
Picton et al. (72); Literature review	Understand effectiveness of outdoor nature based therapeutic recreation programmes from the person with mental illnesses' perspective	*n =* 18 studies		✓		
Ross et al. (73); Review of reviews	Identify the barriers and facilitators to implementing digital health	*n =* 44 studies	✓		✓	
Ross et al. (74); General practice, London, England; Primary study	Imbed T2DM digital health self-management programme into routine care using Normalization Process Theory (NPT)	*n =* 21 staff from *n =* 34 general practices			✓	
Salbach et al. (75); Canada; Primary study	To understand challenges and solutions to implementing community exercise programme model for people with balance and mobility limitations to inform plans for expansion	*n =* 53 stakeholders (healthcare/recreation personnel, programme participants/caregivers; researchers)		✓	✓	
Schmidt et al. (76); Rural Canada; Primary study	Gain a deeper understanding of socio-ecological factors that influence or contribute to physical activity	*n =* 10 older adults aged 69-94			✓	
Sriram et al. (77); Montana, USA; Primary study	Explore how social relationships influence health-related behaviors for people with chronic disease	*n =* 125 midlife and older sedentary overweight/medically obese rural adults		✓	✓	
Stormacq et al. (78); Literature review	Gather best evidence on effectiveness on health-related outcomes of health literacy interventions for enabling socially disadvantaged people living in the community to access, understand, appraise, and apply health information; and identify components of health literacy interventions associated with improved health-related outcomes	*n =* 21 studies	✓			
Sushames et al. (79); Rural and regional Australia; Primary study	Explore perceived enablers and barriers to attendance at an eight-week physical activity programme	*n =* 12 Aboriginal and Torres Strait Islanders	✓			
Taskforce on Multiple Conditions (80); Report	Provide case studies and practical solutions to help local areas improve health and wellbeing for people with multiple conditions	Techniques for change–four common elements	✓	✓	✓	✓
Taylor et al. (81); Remote Canada; Primary study	Explore the experiences and perceptions regarding factors that enable or limit remote videoconference participation, and to obtain suggestions for enhanced delivery of video-conferenced group programs	*n =* 19 stroke survivors (*n =* 12) and caregivers (*n =* 7)			✓	
Wallumbe et al. (82); Rapid review	Identify extent of use of video conferencing technology for delivery of group interventions and provide an overview of its use	*n =* 3 studies; people living with chronic pain	✓			
Zall Kusek and Rist (83); Report	To assist officials, prepare for planning, designing and implementation of results-based M&E.	Provides a handbook for developing, monitoring and evaluating programmes			✓	
		**Total included studies contributing to each theme**	31	14	31	3

*D, development; D, delivery; I, Integration; S, support; T2DM, type 2 diabetes mellitus; QoL, quality of life; HCPs, Healthcare professionals; LTCs, Long-term conditions; COM-B, Capability, Motivation, Opportunity, Behavior. Michie et al. (48)*.

## Results

**Figure 1:**
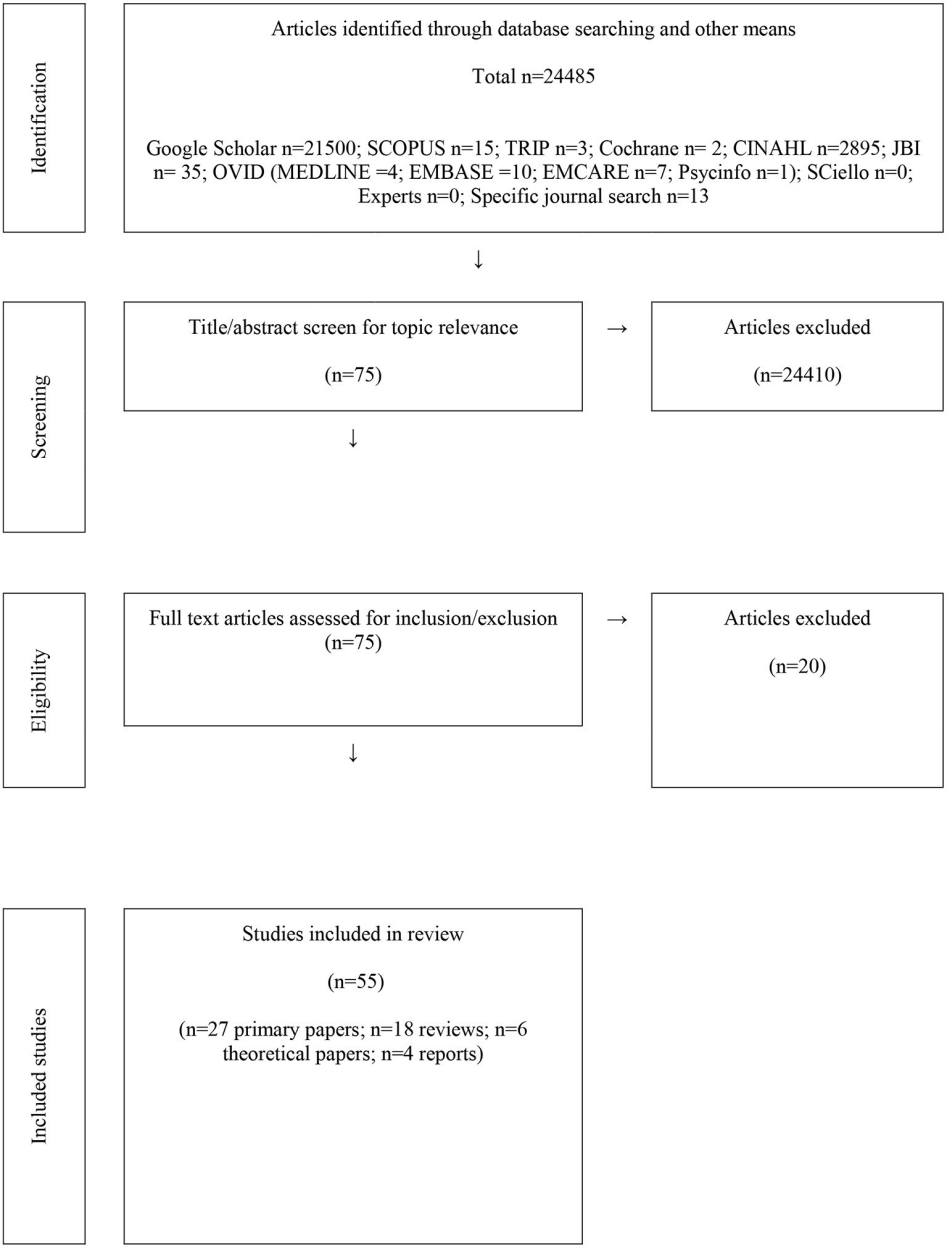
Summary of process of total data through the review.

The search resulted in 24,485 potential articles from which 55 studies were included in the review (Figure 1). A mixture of primary studies (*n* = 27), reviews (*n* = 18), theoretical papers (*n* = 6) and reports (*n* = 4) were included. Five studies related to community-based programmes delivered to a group remotely *via* the internet [Banbury et al., Australia, older persons with chronic disease (33); Del Bello-Haas, Canada, persons with dementia and their carers (41); Jaglal et al., Canada, Chronic Disease Self-Management programme delivered *via* video conference (59); Knox et al., Wales, lung disease (62); Taylor et al., Canada, stroke survivors (81)]. The other 22 primary studies were about in-person delivery of a rehabilitation programme to a group of people. Table 3 also provides an indication of which studies contributed information to the themes.

From the inductive analysis, five categories were derived, theory, context, interpersonal aspects, technology, and programme aspects. Table 4 details the contribution of these categories to the themes. *Development* was the only theme populated by all categories. Only one category contributed to the theme of *Support*. The categories are summarized below.

**Table 4:** Summary of categories contributing to themes.

	**Categories**				
**Themes**	**Theoretical aspects**	**Contextual aspects**	**Interpersonal aspects**	**Technological aspects**	**Programme aspects**
Development	✓	✓	✓	✓	✓
Delivery			✓	✓	✓
Integration	✓		✓	✓	
Support		✓			

### Category 1: Theory

This category contributed information to two themes, “Development” and “Integration”. A framework or theory should be used to both guide development of a rehabilitation programme and its implementation and maintenance (49, 50, 53–55, 64, 70). Use of a theoretical framework makes explicit what the health professional is addressing (66, 70) and thus may also facilitate personal growth for participants (65). Recommended is to develop, with the end-users, goals and a well-defined, efficient (procedures and process, cost), inclusive and adaptable implementation plan (underpinned by an implementation theory) that includes a sustainability plan for the programme/initiative (32, 34, 37, 39, 42, 43, 47, 49, 67, 73). These goals, plans and definitions of success can be identified upfront and need constant reviewing (49, 73). In rural and remote settings, flexibility and creativity are important and need to be utilized in programme design and delivery (42, 71). Focus on outcomes rather than outputs and identify and address barriers (16, 32, 33, 44, 73). Be cognisant of the fact that “one size does not fit all” (67) and that the community needs to want and own the programme or initiative (55, 63, 67–69). Note however, that a community development approach is more time intensive (34, 37, 67, 69).

### Category 2: Context

The category of “Context” contributed information to the themes of “Development” and “Support”. Context is important and collated local knowledge should drive selection of intervention and assessment (47). This necessitates local consultations to find out what people want, need, and prefer (37, 43, 50, 69, 80). Also, of importance is a readiness assessment, for example identification of attitudes to the programme components and intention or readiness to attend (42, 73, 83). A continuing process for identifying and addressing barriers needs to be developed (42, 47, 51, 53, 57, 73, 74, 79, 81). It is important to create an environment whereby attendees become active in managing their own requirements (43). Further, any data collected must be securely stored and privacy is maintained (41, 43, 82). Development of a plan for ongoing infrastructure investment (26, 43, 60) and staff training was emphasized.

### Category 3: Interpersonal Aspects

Three themes, “Development, Delivery and Integration” had contributing information from the “Interpersonal aspects” category. Working *together* on “the project” is essential (33, 34, 39, 69). Create an interactive environment (33) that facilitates development of relationship/social cohesiveness between the participants, spouses, family, and friends (33, 40, 41, 55, 56, 67, 71, 72, 76, 77, 80, 82). Focus on grass roots engagement, identifying shared goals and outcomes, building local resources and networks (67). Ensure projects are community owned and driven, that leadership is representative and inclusive (67). This builds relationships and a collaborative environment that values the contribution of everyone (34). Be aware though that it takes time to learn to work together (53). Build capacity in individuals, groups, and other stakeholders (67). Attract influential members (67). People may need training and require payment for their time (35, 68). Ensure programme is well supported by highly trained staff and volunteers (32, 41, 42, 68, 73). Encourage peer support by using male and female role models/lay leaders (4, 59) and *via* discussion, sharing of stories within the group (33, 81).

### Category 4: Technology

The category of “Technology” contributed information to themes of “Development”, “Delivery”, and “Integration”. Synthesized findings suggest programmes should use technology that is simple, easy to use, adaptable, compatible with existing systems and cost effective (58, 73). Be cognisant of and action regulatory standards, ethics, privacy, security, and storage issues for any data collected (43, 55, 73, 82). Consider use of tools, such as the Universal Design Survey, to assess IT needs/requirements of programme leaders and participants, and train people to use the technology (43). Use creative ways to assist attendees to remember session dates and times (74) and develop telehealth etiquette with them (59). Use innovative ways such as slides and videos to enhance group discussion (33). Plan for interruptions and disconnections to the video feed (35, 81) and hearing issues for attendees (62, 82). Consider where equipment (conferencing and exercise equipment) will be stored (41) and ensure room set up is easy for telehealth and exercise (41, 81). Train the trainers in telehealth etiquette and equipment use, conduct practice teaching sessions (35, 59), and prior to sessions provide a reminder session to review procedures (59) Embed regular monitoring and evaluation (M&E) into all aspects of the programme (49, 66, 73, 83). Involve the team in evaluation and communicate M&E information in multiple ways to stakeholders (49). Link any data collection with existing activities and processes (49, 73).

### Category 5: Programme Aspects

Two Themes, “Development” and “Delivery” had information derived from Category 5: “Programme aspects”. Include/invite/involve people (end-users) in development (33, 34, 39, 69) and provide/create a manual for participants and leaders (46). Address health literacy requirements (4, 55) through use of an ehealth literacy framework (38, 78). Consider use of clinically meaningful assessment and evaluation measures (4), and collection of attendance rates, cost effectiveness (61) and other pertinent data. Include exercise with clear guidance. Advertise the programme in a variety of ways (31, 46), understand and address barriers to attending the programme (42, 47, 51–53, 57, 73, 74, 79, 81), and provide flexibility in programme delivery (e.g., times and places) (35, 42, 51, 55, 64, 79, 85). Need to consider the class size and instructor-to-participant ratio (75) and who will attend, including the minimum level of walking ability, if including physical activity (75). Programme length is recommended to be 4–8 weeks, and use lay and peer led (4) “buddy coaches” with teaching skills to work with the attendees (46).

## Discussion

This integrative review explored literature for international evidence for developing, delivering, sustaining, and supporting a rural or internet delivered, community-wide, generic long-term conditions rehabilitation programme. While the review has several potential limitations (its explorative nature and broad approach, lack of quality appraisal of included studies, and an inherent risk of bias through one author working on inclusion of studies and data extraction), the review nevertheless provides a practical, important and timely contribution to the wider literature. Information gleaned and synthesized from the included studies suggest practical, fundamental points for consideration and were organized into categories of theory, context, interpersonal aspects, technology and programme aspects. The practical implications arising from our findings are summarized in Box 1.

Box 1 **Summary of practical implications arising from findings of the review.**

- Co-development with community end-users should drive intervention and assessment choices and thereby facilitate local ownership of the programme.
- Building local resources, networks, capacity and leadership that is representative and inclusive is important.
- Ensure flexible programme design and delivery.
- Place importance on relationships, social cohesiveness and peer support between attendees, partners, family, and friends, and on highly trained staff and
- volunteers.
- Adopt simple, cost effective technology that is easy to use, adaptable and compatible with existing systems.
- Assess information technology needs of programme leaders and participants, and train people to use the technology.
- Address health literacy requirements.
- Be cognizant of and action regulatory and ethical standards for data collected, plan for interruptions to the video feed, and for hearing issues for attendees.
- Advertise the programme widely and work to understand and address barriers to attendance.

When creating, delivering, sustaining, and supporting a generic rehabilitation programme, the findings from this review suggest the programme needs to be underpinned by “theory.” Such theory is often derived from the field of implementation science (86). Davidoff, Dixon-Woods (87) suggest that while the word “theory” might be an abstract or irrelevant academic term to some, they contend that all people “find and use reasons–and thus theorize” (p. 229) daily. They propose the challenge is to “make explicit the informal and formal theories” (p. 230) people use because this may highlight assumptions, weaknesses, or contradictions in the proposed intervention programme's hypothesis, and expose any lack of consensus among the team (87). Use of what is termed a “small theory” or “programme theory” provides a framework for outlining programme components, expected outcomes and their assessment methods (87). Additionally the theory assists to make explicit and clear the assumptions and rationale linking “processes and inputs to outcomes … and conditions (or context) necessary for effectiveness” (p. 230) (87). Many people skip working out the programme theory and rush to implementation, thus limiting “learning that can inform planning of future interventions” (p. 232) (87). Choosing a theory may not be that straight forward. Lynch, Mudge (88) and Nilsen (86) in their debate papers provide useful summary for understanding available theories (current at time of publication of their papers), and a starting point and pragmatic guide for selection of “theory/ies” to underpin programmes/interventions.

This review highlighted the importance of interpersonal factors for developing, delivering, sustaining, and supporting a programme. Working together with the people to whom it matters on programme development requires time to build relationships, talk, acknowledge and share power, reflect, and return repeatedly to these processes as the programme is developed, delivered, and evaluated (89, 90). Time that is often not always available in the research arena because of constraints applied by funders and commissioners, or even because of a difference in world views between team members and community members (a biomedical v a bio-psycho-social viewpoint). While time may not be “available,” relationships are integral to care and healing processes (91). Development and maintenance of meaningful relationships with other people is acknowledged to lead to improvement in wellbeing and health (92–95). The concept of relationship-centered care, argued to be the founding principle for all healthcare provision (91), may provide a framework for understanding the interrelated relationships necessary when working on programme development together with people to whom it matters.

Linked with the importance of developing meaningful relationships and working together with stakeholders (individuals, groups, communities, policy makers) are issues of pertaining to the context, particularly of valuing local knowledge about what is wanted, needed, and preferred. For developers, there are many ways to approach this depending on the philosophical and methodological viewpoint. For example, in included studies where the programme developers have already defined the topic of interest, to a study where the developers join with a community of stakeholders, and the community discuss what needs to be explored (96) (using a Participatory Action Research or co-design approach). Such stakeholder involvement can range from defining the issue/s, developing the programme, through to contributing as a participant, or interest only in the outcomes of the programme development project (97). Boaz, Hanney (97) suggest the literature assessing the impact of stakeholder engagement is limited but an increasing area of interest. They put forward three design principles for stakeholder engagement of organizational, values, and practices (with supporting literature) for developers to consider when thinking about stakeholder engagement and promoting impact of project development (97).

The idea of assessing readiness for change/engagement by people, communities, and organizations would also seem useful. Yet terminology used in the area is confused, and there is no gold standard assessment available as instruments available are tailored to specific contexts and/or interventions (98). Miake-Lye, Delevan (98), in their systematic review of organizational readiness assessments mapped to the Consolidated Framework for Implementation Research (CFIR), suggest the seven most frequent CFIR constructs identified (readiness for implementation, implementation climate, other personal attributes, structural characteristics, networks and communications, self-efficacy, and culture) could provide something to consider when developing or tailoring a readiness assessment. Miake-Lye, Delevan (98) made only minor amendments to classify items, suggesting readiness for change is captured in the CFIR, with one addition relating to teams. It seems using a broad framework such as the CFIR may be another useful tool for programme developers.

The main findings from the review regarding “Technology” highlight attention to the principles of “KISS” (keep it simple stupid) (99) and Universal Design (100, 101), and integration of health and eHealth literacy concepts (38) across all phases of implementation of the programme to facilitate access to the programme for a wider range of people. Additionally, the importance of embedding monitoring and evaluation processes within all phases of programme “development” seems pertinent to assess effectiveness of an intervention.

Many of the practical tips embedded in “Programme aspects” reiterate the importance of attending to the interpersonal factors discussed above. Further findings highlighted the importance of addressing health literacy requirements of participants. Health literacy is important and much has been written about it (78, 102–105). However, “health literacy” is greater than individual competencies alone, it also includes community, services, and health system literacy capacities (106) and all these areas need to be considered and addressed when developing a programme or intervention.

## Conclusion

Our review has drawn together a large body of diverse work. It has focused on finding practical information pertaining to the best ways to develop, deliver, integrate, and support a community-based group generic rehabilitation programme for people living with long-term health conditions, delivered rurally in person and/or potentially *via* the internet. Practical suggestions were thematically organized into categories of “theory”, “context”, “interpersonal aspects”, and “technology” and “programme aspects”. Box 1 provides a summary of the practical implications derived from the review. While the findings of this review might appear simple and self-evident, they may be difficult to enact in practice.

## Author Contributions

Conception and design: LH, TS, CH, and JD. Data collection: AW. Data analysis and interpretation and drafting original article: AW and LH. All authors critical revision of article and final approval of version to be published.

## Funding

This work was supported by the Health Research Council of New Zealand [Grant Number 20/1167].

## Conflict of Interest

The authors declare that the research was conducted in the absence of any commercial or financial relationships that could be construed as a potential conflict of interest.

## Publisher's Note

All claims expressed in this article are solely those of the authors and do not necessarily represent those of their affiliated organizations, or those of the publisher, the editors and the reviewers. Any product that may be evaluated in this article, or claim that may be made by its manufacturer, is not guaranteed or endorsed by the publisher.
